# Perioperative outcome of primary total hip arthroplasty in octogenarians – A systematic review

**DOI:** 10.1016/j.jor.2024.11.001

**Published:** 2024-11-02

**Authors:** Annemarie Rusche, Georg Osterhoff, Andreas Roth, Nikolas Schopow

**Affiliations:** Department for Orthopedics, Trauma Surgery and Plastic Surgery, University Hospital Leipzig, Leipzig, Germany

**Keywords:** Primary total hip arthroplasty, Total hip arthroplasty, Octogenarians, Elderly

## Abstract

Osteoarthritis (OA), a degenerative disease, frequently necessitates the replacement of the affected joint, commonly involving the hip. With increasing life expectancy and the anticipated rise in OA incidence, the demand for Total Hip Arthroplasty (THA) is expected to surge, particularly among Octogenarians. The elderly are often perceived as a vulnerable patient group, with concerns about their eligibility for THA primarily based on age alone.

**Aims:**

There is a prevailing presumption that Octogenarians face an elevated risk of perioperative mortality, increased complication rates, and prolonged hospital stays.

This systematic review aimed to compare perioperative outcomes after THA in Octogenarians, with primary outcomes being mortality and length of stay in hospital.

**Methods:**

A systematic screening of 33,336 titles from the PubMed and Web of Science databases following Cochrane Handbook for Systematic Reviews and PRISMA guidelines was performed. Eight publications about THA met inclusion criteria for final analysis, Studies underwent evaluation for certainty of evidence according to GRADE criteria.

**Results:**

Eight studies were included in this systematic review, covering data from 494,144 patients.

Perioperative mortality rates varied between 0.33 % and 1.96 % in the older cohort. The duration of hospital stay was longer among Octogenarians compared to their younger counterparts across all publications. Surgical complications were similar in elderly and younger patients. Although elderly patients generally experienced higher rates of medical complications, differences were subtle.

**Conclusion:**

Age alone should not hinder performance of THA in Octogenarians. This systematic review underlines that eligibility for a procedure should be based on comorbidities and overall health status. Important findings from international guidelines for improving safety in elderly undergoing THA were included, but further research is needed to ensure optimal perioperative management of risk factors and potential complications.

## 1 Introduction

Osteoarthritis (OA) stands as one of the foremost global causes of disability, affecting a substantial proportion of the population. According to the Global Burden of Disease 2019 study, osteoarthritis is affecting 7.6 % of the world population.^1^ Osteoarthritis is an inflammatory disease that involves complex interactions of the cartilage, subchondral bone, and synovium. The result is a chronic inflammation and consequently the destruction of the joint, leading to severe pain. Increasing age and obesity are important risk factors for OA.^1^^,^^2^

The treatment is multimodal and involves conservative treatments, e.g. with medication and physiotherapy but also surgical treatments, such as the total joint replacement.^2^ According to the Organization for Economic Cooperation and Development

(OECD), an average of 174 THAs per 100,000 inhabitants were performed per year in 2019.^3^ The Global Burden of Disease 2021 projects that in the next 30 years, there will be 78.6 % more cases of hip OA^1^ This increase is attributed to the aging of population and the rising prevalence of obesity.^1^^,^^2^ Due to high frequency of this procedure, it is necessary to investigate the consequences it might have when performed on older patients. Octogenarians, individuals aged over 80 years, are often considered a fragile patient group. According to literature, increasing age is an important risk factor for post-surgical morbidity and mortality.^4^^,^^5^ Elderly often experience substantial benefits from THA, with improvement in functional outcomes, satisfaction and quality of life, comparable to those seen in younger patients.^6^ It is also worth considering the cost-effectiveness of THA in this age group when compared to conservative treatments.^7^ No meta-analyses or systematic reviews on this topic have been conducted in the past years. The aim of this systematic review is to investigate the perioperative outcomes of THA performed on Octogenarians in comparison to a younger control group. Additionally, this review seeks to compile existing data, emphasize the need for prospective studies, and promote further research.

## 2 Methods

**Fig. 1:**
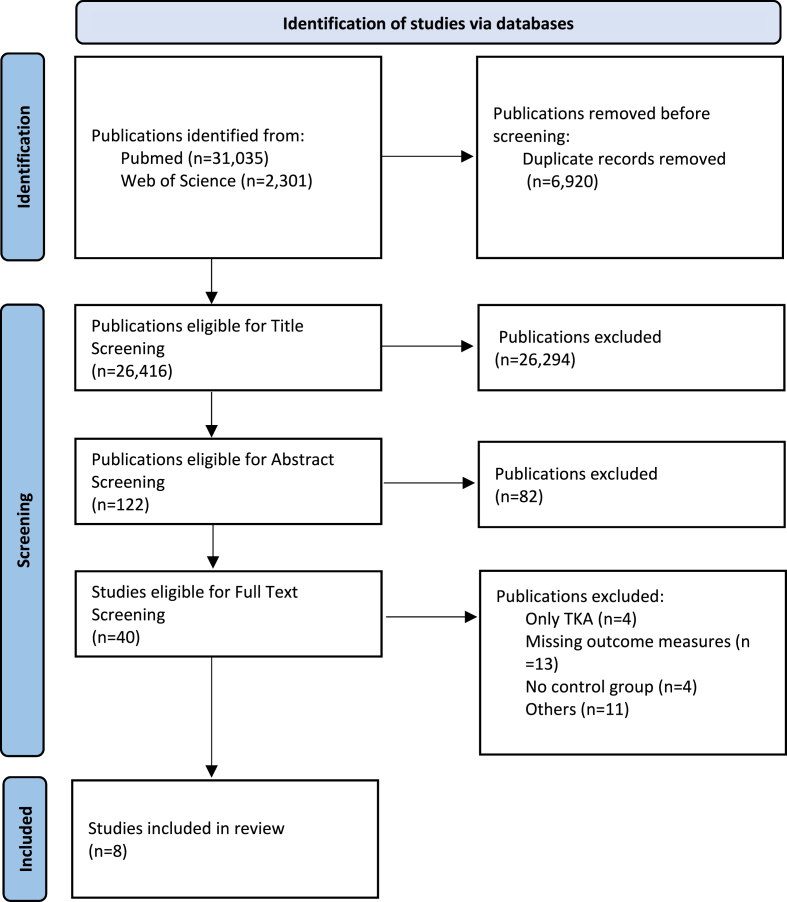
PRISMA flow diagram for systematic reviews. This figure illustrates the method used for screening according to PRISMA guidelines. After identifying 33,336 publications, eight studies were included for review.

This review followed PROSPERO registration (CRD42022337168) and adhered to the Cochrane Handbook for Systematic Reviews of Interventions and the PRISMA guidelines.^8^^,^^9^ Databases used for research were PubMed and Web of Science, last updated on June 22, 2024, using the search terms: ((Total hip arthroplasty OR THA) OR (total knee arthroplasty OR TKA)) AND (geriatric OR elderly OR octogenarian). Language filters (English, German, and French) were applied and a publication date (from January 1, 2012, to May 31, 2024) was set. Two reviewers independently identified relevant studies. Inclusion and exclusion criteria were established using the PICOS framework: studies had to focus on Octogenarians (average age 80+) undergoing primary THA. A younger control group was necessary for comparison. Main outcomes included mortality, hospital stay duration, and surgical and medical complications. Eligible study types were randomized controlled trials (RCTs) and clinical trials. Articles without full texts, case reports, conference communications, systematic reviews, meta-analyses and non-primary procedures (e.g. post-trauma or revision surgeries) were excluded. Our search yielded 33,336 publications, further details of which are in the PRISMA 2020 flow diagram (Fig. 1). Data from PubMed and Web of Science were analyzed using Zotero and Excel. Main outcomes (mortality and hospital stay duration) underwent Grading of Recommendations, Assessment, Development and Evaluation (GRADE) and Risk Of Bias In Non-randomized Studies - of Interventions (ROBINS-I) assessment.^8^^,^^10^ The GRADE criteria evaluate certainty of evidence in systematic reviews, considering the following key points: study design, risk of bias, inconsistency, indirectness, imprecision, and publication bias, ranking from high to very low.^8^ Risk of bias was assessed using ROBINS-I tool, consisting of seven components: confounding, participant selection, intervention classification, adherence, missing data, outcome measurement, and result reporting. Bias is rated from low to critical.^10^ The Oxford Centre for Evidence-Based Medicine (OCEBM) 2011 criteria were used to determine evidence levels.^11^ OCEBM Levels of Evidence ranks medical evidence quality from Level 1 (systematic reviews, randomized controlled trial) to Level 5 (expert opinion). Request for additional data from the authors of the studies primarily included was made but did not yield any further information. No extra publications were found through reference list screening.

## 3 Results

33,336 publications were found by searching according to the predefined criteria in both databases. Following the elimination of 6920 duplicates, 26,416 publications were subject to title screening. Abstract screening was performed on 122 publications, resulting in 40 remaining. Following full-text screening, eight publications examining perioperative outcomes in THA were included in this systematic review. Data of 494,144 patients receiving THA between 1994 and 2021 were analyzed, with the largest set of data being from the study performed by Leopold et al. including 263,967 patients.^12^ Five studies included information obtained from registry databases.^12^^,^^15^, ^16^, ^17^, ^18^ Leopold et al. conducted a registry study using data from the German Arthroplasty Registry (EPRD), finding a mean age of 82.7 years in the OC compared to 74.8 years in the YC. Patients in the OC were more likely to be female (70.9 % vs. 66.9 %). Mortality rates were evaluated one-year post-surgery.^12^ Kurapatti et al. focused on the oldest patients, with a mean age of 91.6 years in the OC, using 1:1 propensity score matching based on Body Mass Index (BMI), American Society of Anesthesiologists (ASA) score, and Charlson Comorbidity Index (CCI).^13^ Zak et al. argued that certain demographic characteristics, such as higher ASA score, are commonly found in elderly patients and did not match based on these factors to create a representable OC.They reported the largest age gap between the OC and YC (mean age 84.3 years vs. 61.6 years).^14^ Sherman et al. sourced data from the American College of Surgeons National Quality Improvement Program (ACS NSQIP) and performed matching on demographics and comorbidities.^15^ Boniello et al. also analyzed ACS NSQIP data, noting that the OC had a significantly lower mean BMI than the YC (27.0 kg/m^2^ vs. 30.3 kg/m^2^).^16^ Murphy et al. drew data from the Australian Arthroplasty Registry, showing the OC had a higher representation of women, a lower BMI, and a higher ASA score compared to the YC.^17^ Miric et al. used the Total Joint Replacement Registry (TJRR). The OC had higher rates of females (67.9 % vs. 56.2 %) and a significantly lower mean BMI (26.4 kg/m^2^ vs. 29.6 kg/m^2^).^18^ In the study by Kennedy et al. the OC had a mean age of 84 years compared to 66 years in the YC, with no matching performed.^19^ More detailed information about patient characteristics is provided in Table 1.

**Table 1:** Characteristics The table displays the characteristics of the studies and patients. Number of included patients and data collection period are presented. Data was sourced from either single-center, multi-center studies or registry studies. Mean age, proportion of female patients and Body Mass Index (BMI) within cohorts are displayed. OC: Octogenarian cohort; YC: younger cohort; SD: standard deviation; N/A: data not available; n: Number of Patients a no further information to “prevent patient identification”^15^ b no further information available.

	n	Data collection period	Study Type	Mean age (±SD)		Female gender in %		Mean BMI (±SD)	
				OC	YC	OC	YC	OC	YC
**Leopold (2023)**	263,967	2012–2021	Registry	82.7 ± 2.41	74.8 ± 2.83	70.9	66.9	N/A	N/A
**Kurapatti (2022)**	14,824	2011–2021	Single-center	91.6 ± 1.7	75.8 ± 7.9	59.3	63.0	24.9 ± 4.4	25.2 ± 4.9
**Zak (2021)**	10,860	2011–2019	Single-center	84.3 ± 3.0	61.6 ± 10.7	67.6	55.2	26.9 ± 4.4	29.7 ± 49.5
**Sherman (2020)**	88,058	2008–2017	Registry	>90^a^	74.5 ± 6.6	71.9	70	25.9 ± 6.6	26.5 ± 6.1
**Boniello (2017)**	66,839	2011–2014	Registry	83.4 ± 2.6	62.5 ± 10.2	55	66	27.0 ± 5.9	30.3 ± 6.9
**Murphy (2017)**	2139	2006–2014	Registry	≥80^b^	<80^b^	67.0	59.0	30.0 ± 4.7	30.5 ± 6.0
**Miric (2015)**	43,543	2001–2011	Registry	83.0	63.3± N/A	67.9	56.2	26.4 ± 4.6	29.6 ± 5.9
**Kennedy (2013)**	3914	1994–2004	Multi-center	84 ± N/A	66 ± N/A	≈67.8	≈57,8	N/A	N/A

Perioperative mortality rates, assessed between 30 or 90 days postoperatively ranged between 0.33 % and 1.96 % in the OC vs. 0.02 %–1.9 % in the YC.^13^, ^14^, ^15^, ^16^, ^17^, ^18^, ^19^ The highest mortality rates were observed in the study by Leopold et al. where mortality was assessed one year post-surgery, reporting rates of 11.8 % in the OC and 6.0 % in the YC.^12^ A significantly higher mortality rate among Octogenarians was reported by five studies.^12^^,^^14^^,^^16^, ^17^, ^18^ Two studies observed higher perioperative mortality among the elderly cohort, but these rates were not significantly higher compared to the YC.^15^^,^^19^ Interestingly, the study by Kurapatti et al. found no difference in the 90-day mortality rate between matched cohorts.^13^ Octogenarians experienced prolonged hospital stays across all studies.^12^, ^13^, ^14^, ^15^^,^^17^, ^18^, ^19^ Five studies observed significantly longer LoS in the OC compared to the YC.^13^, ^14^, ^15^^,^^18^^,^^19^ In five studies, LoS ranged from 3.3 to 6.4 days vs. 2.5–5.2 days.^13^, ^14^, ^15^^,^^17^^,^^18^ The certainty of evidence concerning the outcome measures of mortality and LoS was assessed using GRADE criteria, and both outcomes were rated as having 'very low' evidence. For further information, see Table S1.

Complication rates, surgical and medical, varied across studies and were highly heterogenous. Three studies evaluated overall surgical complication rates, reporting similar rates in the two cohorts.^13^^,^^17^^,^^19^ One study additionally assessed wound complication rates and found no difference between the two cohorts.^17^ Surgical Site Infections, including Periprosthetic Joint Infection, Superficial Infection, Deep Infection and Wound Infection, were examined in seven studies. Three studies observed higher infection rates in the YC, while four studies reported similar or slightly higher infection rates in the OC.^12^, ^13^, ^14^, ^15^, ^16^^,^^18^^,^^19^ Periprosthetic and femoral fracture were documented in three studies. One study reported similar rates of fracture in both cohorts, while two studies found higher fracture rates in the YC.^12^^,^^13^^,^^19^

Overall medical complication rates were assessed in three studies, with two indicating higher rates in the OC and one showing comparable rates between the two cohorts.^13^^,^^17^^,^^19^ One study separated major and minor adverse events, both appear more frequently in elderly.^12^

Three studies examined anemia and bleeding requiring transfusion, observing higher rates in the OC.^12^^,^^16^^,^^19^ Three studies found myocardial infarction and cardiovascular complications to be more prevalent in the YC.^13^, ^14^, ^15^ In three different studies, these complications were primarily seen in elderly patients.^12^^,^^16^^,^^19^ Pulmonary embolism rates were higher in the elderly in four studies, while one study found it more common in the YC.^12^^,^^15^^,^^16^^,^^18^^,^^19^ Urinary tract infections were consistently higher in the OC across three studies.^15^^,^^16^^,^^19^ Pneumonia and chest infections were reported in four studies, with two showing higher rates in elderly, while one reported similar rates, and another study found more chest infections in the YC.^12^^,^^15^^,^^16^^,^^19^ Confusion and delirium were reported in two studies, both documenting higher rates in elderly.^12^^,^^19^ Further details regarding outcome measures are presented in Table 2.

**Table 2:** Mortality, Length of stay and Complications The table outlines the primary outcomes, including mortality, length of stay (LoS), and Complication rates in the Octogenarian cohort (OC) and younger cohort (YC). Mortality rates were evaluated after 30 or 90 days, except for Leopold et al. providing 1year mortality rates. Complications were categorized into surgical and medical, with the three most common complications, if available. SD: standard deviation; p: statistical significance; N/A: data not available; PJI: Periprosthetic Joint Infection; PPF: Periprosthetic fracture; CKD: Chronic Kidney Disease; MI: Myocardial infarction; UTI: Urinary tract infection.

	Mortality in %			Mean LoS in days (±SD)			Complications in %							
	OC	YC	p	OC	YC	p	Surgical complications	OC	YC	p	Medical complications	OC	YC	p
**Leopold (2023)**	11.80	6.00	N/A	10.6 ± 7.60	9.29 ± 8.00	N/A	PPF PJI	1.00 0.50	0.90 0.40	N/A N/A	Anemia Exacerbation of CKD Electrolyte disorder	28.70 14.80 9.80	20.70 8.90 6.40	N/A N/A N/A
**Kurapatti (2022)**	1.90	1.90	1.00	3.90 (±2.1)	3.10 (±1.9)	0.031	PPF PJI	0.00 0.00	1.90 1.90	N/A N/A	Cardiovascular Complications Others	0.00 1.90	1.90 1.90	N/A N/A
**Zak (2021)**	0.33	0.02	0.001	3.50	2.50	<0.001	PJI Dislocation Aseptic Loosening	0.30 0.00 0.00	0.86 0.14 0.00	0.214 0.993 0.993	MI	0.00	0.01	0.993
**Sherman (2020)**	0.90	0.70	0.59	3.52 ± 2.81	2.81 ± 1.87	<0.001	Surgical site Infection	0.60	0.30	0.73	UTI Pneumonia MI	2.80 1.50 0.7	0.90 1.00 1.0	0.004 0.39 0.44
**Boniello (2017)**	0.90	0.10	<0.001	N/A	N/A	N/A	Superficial Infection Deep infection Wound Infection	0.80 0.20 0.30	0.70 0.20 0.30	0.30 0.28 0.22	Bleeding requiring Transfusion UTI Pneumonia	24.80 2.70 0.90	12.50 1.00 0.30	<0.001 <0.001 <0.001
**Murphy (2017)**	1.90	0.20	0.001	6.37 ± 3.51	5.17 ± 2.43	N/A	Wound Complications Others	14.10 4.00	11.60 3.60	N/A N/A	Total	27.60	9.50	N/A
**Miric (2015)**	0.70	0.10	N/A	3.3 ± 1.8	2.8 ± 1.5	N/A	Superficial Infection Deep Infection	0.50 0.50	0.30 0.50	N/A N/A	Deep Vein Thrombosis, Pulmonary Embolism	1.20 0.60	0.70 0.50	N/A N/A
**Kennedy (2013)**	1.60	0.30	N/A	12.00	9.00	<0.001	Dislocation Femoral Fracture Superficial Infection	3.10 2.10 1.90	3.90 3.30 2.10	N/A N/A N/A	UTI Chest Infection Confusion	3.10 1.80 2.90	1.70 2.20 0.60	N/A N/A N/A

Surgical complications were observed at similar rates in both cohorts. While medical complications were generally more frequent in elderly patients, many of these showed only marginally differences. Anemia, transfusions, urinary tract infections, and confusion and delirium were generally observed more frequently in elderly patients.

## 4 Discussion

Increasing life expectancy and other factors, such as obesity and diabetes are leading to an increase in osteoarthritis, which currently affects 518 million people worldwide, with a simultaneous increase in the proportion of patients for THA with relevant pre-existing conditions.^20^ Therefore, the aim of this systematic review was to investigate the influence of advanced age on perioperative outcome after this procedure. We could show that THA can safely be performed on Octogenarians if they are eligible for the procedure. Age should not be an exclusion criterium.

### 4.1 Mortality

In the literature, advanced age has been associated with higher mortality rates.^4^^,^^24^ This finding is partly reflected in our review, since mortality rates were higher in the OC in seven of eight studies.^12^, ^13^, ^14^, ^15^, ^16^, ^17^, ^18^, ^19^ However, the difference in perioperative mortality between the cohorts was found to be minimal.^13^, ^14^, ^15^, ^16^, ^17^, ^18^, ^19^ The risk of undergoing surgery needs to be thoroughly evaluated. Elderly patients, especially with preexisting comorbidities, should be informed about the increased risk of postoperative mortality. However, it is equally important to emphasize that overall mortality rates remain relatively low, and the potential benefits of the procedure, improvements in functionality and quality of life, should also be considered in the decision-making process.^21^^,^^22^ Preoperative mortality risk should be evaluated using scoring systems such as the Clinical Frailty Scale.^23^ Assessing risks of postoperative mortality can enhance informed consent and shared decision making. Additional clinical practice recommendations for performing THA in elderly patients are summarized in Table 3.

**Table 3:** Tips and tricks for elderly undergoing THA This table presents potential enhancement for optimal outcomes of THA in elderly. Data was derived from international guidelines and current literature. The right column provides information about the Strength of Recommendations, Levels of Evidence (LoE) and Consensus-based Evidence. ∗Strength of option, since little evidenced-based data is available. CFS: Clinical Frailty Scale; BMI: Body Mass Index; LoS: Length of Stay, NSAID: Nonsteroidal Anti-Inflammatory Drugs; Hb: Hemoglobin; MMSE: Mini-mental State Examination.

Phase of THA	Explanation	Strength of Recommendation/LoE/Consensus-based Recommendation
**Indication**	Clinical Diagnosis “Osteoarthritis” (anamnesis and clinical test)^41^	−/−/Strong consensus
	Non-medical treatment e.g. education, exercise therapy, and weight reduction, should be pursued before considering THA^41^	Strong/1+/Consensus
	Indication for THA should be considered (after conservative treatment) in patients with persistent pain, impairment of function, activities of daily life and health related quality of life^41^	Strong/3/Strong consensus
	Osteoarthrosis with minimum Kellgren/Lawrence Grade 3 in radiological imaging^41^	−/−/Strong consensus
	Intraarticular injection of corticosteroids could be considered to improve short-term function and reduce pain^42^	Strong/−/−
	Preoperative mortality risk can be evaluated using scores, e.g. CFS, for improved shared-decision making^23^	−/−/Recommended in literature and by the authors
**Modifiable Risk Factors**	Anemia diagnostics and treatment^41^	-/1-or 2+/Consensus
	HbA1c <8 %^41^	Strong/2++/Strong consensus
	Weight reduction when BMI ≥30 kg/m^2^^41^	Moderate/-/
	Preoperative multidisciplinary care provision especially for complex patients to improve postoperative LoS^43^	−/−/Recommended in literature and by the authors
**Perioperative Management**	Cemented fixation could be considered due to lower risk of Periprosthetic Fracture^42^	Moderate∗/-/
	Preoperative or early postoperative use of NSAIDs reduces pain and opioid use^44^	Strong/1/-
	Transfusion should be considered in elderly (>65years) undergoing trauma- or orthopaedic surgery with Hb < 8 g/dl^45^	Strong/1/-
	Tranexamic acid to reduce blood loss and need for transfusion^42^	Moderate/-/
	Prevention of Delirium, including assessment of cognitive functions (e.g., MMSE), evaluation of risk factors, adequate pain management, adequate sleep, hydration and nutrition, cognitive stimulation, and correction of sensory deficits (e.g., hearing and eyesight)^46^	−/−/Recommend in literature and by the authors
**Postoperative Management**	Postoperative Physical Therapy^42^	Moderate/-/
	Early Joint mobilization postoperatively^44^	−/−/Recommend in literature and by the authors

### 4.2 Length of stay

Octogenarians had a longer hospital stay throughout all studies.^12^, ^13^, ^14^, ^15^, ^16^, ^17^, ^18^, ^19^ This finding aligns with literature.^4^^,^^24^ In six studies, mean LoS in the OC was circa one day longer compared to the YC (0.5–1.3 days).^12^, ^13^, ^14^, ^15^^,^^17^^,^^18^ Therefore, prolonged LoS in elderly may not be clinically relevant.

Two studies did observe considerably longer mean LoS in both cohorts, compared to the others.^12^^,^^19^ Kennedy et al. reported a mean LoS of twelve days in the OC and nine in the YC, which may be associated to the study period, beginning in 1994, and a trend towards shorter LoS in the last decades.^19^^,^^25^ In the UK, patient's’ hospital stay decreased from 10.7 to 6.8 days during the same period. Leopold et al. and suspects that comorbidities are more frequent among the OC, thus resulting in longer LoS, which has been reported in literature as well.^12^^,^^26^ A longer hospital stay usually generates higher costs and can be an important economic burden for the healthcare system.^27^ A longer hospital stay does not have to be a disadvantage for patient and hospital, though. A study examined the impact of ortho-geriatric co-management on mortality rates following femoral neck fracture. Patients receiving early ortho-geriatric rehabilitation stayed 5.4 days longer in hospital than those in the comparison group but had a 22 % reduction in 30-day mortality rates.^28^

### 4.3 Perioperative complications and risk factors

A wide range of complication rates is presented throughout this systematic review. No consistent trend can be identified for different types of complications. In current literature, ASA score and comorbidities were identified as independent risk factors for complication rates after trauma and general surgery.^29^, ^30^, ^31^, ^32^ A statistical comparison between cohorts, using ASA score and comorbidity index as confounders for postoperative complications was not possible due to different approaches used for matching and lack of data. Surgical complications were similar between cohorts in all studies. Evidence suggests that elderly patients are more prone to medical complications. Certain complications, e.g., anemia, need for transfusion, urinary tract infections, and delirium and confusion, are particularly relevant in the elderly population. However, the overall incidence of most complications remained similar between cohorts. This finding may be influenced by selection bias, as only elderly patients deemed eligible for surgery were included in these retrospective studies. Further exploration of protective factors in the elderly.

### 4.4 Pre- and postoperative management

The influence of prehabilitation and rehabilitation programs on postoperative outcomes is not to be underestimated. In the study designed by Kurapatti et al. patients underwent a standardized rehabilitation program.^13^ Specific rehabilitation adapted to the needs of elderly can be one variable to further minimize postoperative surgical complications.^33^ Fast track rehabilitation offers an alternative approach to minimize hospital stays and improve functional outcomes postoperatively, without increasing the risk of complications.^34^ Early rehabilitation programs, adapted to the special needs of this potentially vulnerable patient group, might be able to minimize postoperative complications and reduce costs in the long term.

### 4.5 Surgical techniques and approaches

Discrepancies in intraoperative observations might have an influence on postoperative outcome. There is no clear indication for longer operation time, but since it is associated with more blood loss during surgery, postoperative anemia and need for transfusion, it should be kept as short as possible.^35^ Mortality rates are similar whether using cement free or cemented implants in total hip replacements.^36^ Use of cemented implants can lead to perioperative complications, like bone cement implant syndrome (BCIS) or pulmonary embolism.^37^ Preventative measures for reducing risk of cement embolism are important, especially in older patients.^38^ But, revision rates were higher with cement free implants, mostly because of periprosthetic fractures.^39^ Therefore, it is recommend to use cemented implants in older patients, especially in woman aged above 75 years.

### 4.6 Limitations

There are some limitations to this systematic review. Few studies compare Octogenarian cohorts to control groups. Eligible studies often report different outcome measures, complicating direct comparisons. Data were systematically sourced from only two databases, which may result in the omission of important articles. Other databases were considered but deemed unsuitable or inaccessible. Although a meta-analysis was considered, it was ultimately deemed unfeasible due to heterogeneity and insufficient data. Another aspect to consider is that certain studies were matched 1:1 for various patient characteristics, such as BMI, comorbidities, or ASA score. However, this approach may introduce confounding bias, as these characteristics have been identified as potential confounders for perioperative outcome measures. Furthermore, it may create an unrepresentative cohort with characteristics, unlikely for the average elderly. A limitation arises from the lengthy data collection period across studies, with data spanning from 1994 to 2021. Changes in surgical techniques and advancements in implant technology during this time may introduce confounding factors that affect the comparability of outcomes. The term complication is heterogeneously defined among authors and further analysis was not possible. Moreover, because of the retrospective nature of the study design and data analysis, determining the exact incidence of postoperative complications might prove challenging. This difficulty arises from the fact that not all minor complications may be documented years after the hospital stay. Two outcomes, mortality and LoS were further analyzed regarding their quality of evidence. Data must be considered highly biased and therefore low quality of evidence. Especially pre-intervention bias domains, e.g., confounding and selection of participants, had great impact on overall risk of bias assessment. An important factor contributing to the assessment of overall evidence as very low is the inconsistency of results. For instance, regarding mortality rates, even when examining the same time period mortality rates exhibit significant variation across studies. This discrepancy has been described in previous literature, where predicted 30-day mortality varied between 0.05 % and 6.55 %, according to differences in age, sex and patient frailty.^40^ Data regarding duration of hospital stay is even more inconsistent. This makes it difficult to compare and evaluate data from all studies included.

### 4.7 Conclusion

More research is needed to provide optimal patients safety for this common procedure. Prospective randomized controlled trials are warranted to explore additional potential risk factors. Furthermore, factors influencing optimal pre-, intra-, and postoperative patient management should be investigated to ensure patients safety. There are indicators, that comorbidities are more relevant for surgery outcome than age. Furthermore, institution and hospital should emphasize on ortho-geriatric pre- and rehabilitation to ensure ideal surgery outcomes. In summary, our findings suggest that comorbidities and overall health status independently contribute to higher mortality rates, prolonged hospital stays, and perioperative complications.

## Conflict of interest

The authors received no external financial or material support for the research, authorship, and/or publication of this article.

## CRediT authorship contribution statement

**Annemarie Rusche:** Conceptualization, Methodology, Formal analysis, Investigation, Writing – original draft, Data curation. **Georg Osterhoff:** Conceptualization, Writing – review & editing, Supervision, Project administration. **Andreas Roth:** Conceptualization, Writing – review & editing, Supervision, Project administration. **Nikolas Schopow:** Conceptualization, Validation, Writing – review & editing, Visualization, Supervision.

## Patient's consent

This research did not require patient's consent because we did a retrospective analysis of data already published.

## Ethical statement

Ethical approval was not sought for the present study because retrospective data was used, which was already published.

## Funding statement

The authors received no external financial or material support for the research, authorship, and/or publication of this article.
