# Supplementary material for: Perioperative outcome of primary total hip arthroplasty in octogenarians – A systematic review
Source: J Orthop. 2024 Nov 2;60:152–8. doi: 10.1016/j.jor.2024.11.001 (PMC11602539; doi:10.1016/j.jor.2024.11.001)
Supplement: Multimedia component 2 [file mmc2.docx]

| **Outcomes**  **N of patients**  **(studies)** | **Effect estimates** | **Certainty of Evidence (GRADE)** |
| --- | --- | --- |
| **Mortality**  **N=404,377**  **(8)** | Octogenarian Cohort vs. Younger Cohort  Not possible | Very low  Due to Risk of bias and Inconsistency |
| **Length of stay**  **N=337,538**  **(7)** | Octogenarian Cohort vs. Younger Cohort  Not possible | Very low  Due to Risk of bias and Inconsistency |

Table S1 - Summary of Findings Table (SoF)

This table displays rating for Certainty of Evidence for two main outcomes: mortality and Length of Stay. The absence of relative effects in this SoF arises from the inherent incompatibility of the specific data presented in the chosen studies. All the studies used are retrospective cohort studies, therefore risk of bias is very likely to appear. Especially factors influencing risk of bias pre-procedure, e.g. due to confounding or selection of patients led to downgrading by at least one level. Downgrading by one level was done since all studies are retrospective and there is a chance of selective outcome measurement and reporting. Effect estimates could not be generated due to limitations in the available data, e.g. lack of sufficient comparable studies, significant heterogeneity in study designs, or incomplete reporting of outcome measures. As a result, conducting a meta-analysis was not feasible, and no pooled effect estimates are presented.
